# What we talk about when we talk about COVID-19 vaccination campaign impact: a narrative review

**DOI:** 10.3389/fpubh.2023.1126461

**Published:** 2023-05-11

**Authors:** Horácio N. Hastenreiter Filho, Igor T. Peres, Lucas G. Maddalena, Fernanda A. Baião, Otavio T. Ranzani, Silvio Hamacher, Paula M. Maçaira, Fernando A. Bozza

**Affiliations:** ^1^Department of Industrial Engineering, Pontifical Catholic University of Rio de Janeiro, Rio de Janeiro, Brazil; ^2^School of Management, Federal University of Bahia, Salvador, Brazil; ^3^Barcelona Institute for Global Health, Barcelona, Spain; ^4^Pulmonary Division, Heart Institute, Faculty of Medicine, Hospital das Clínicas da Faculdade de Medicina da Universidade de São Paulo, São Paulo, Brazil; ^5^National Institute of Infectious Disease Evandro Chagas, Oswaldo Cruz Foundation, Rio de Janeiro, Brazil; ^6^D'Or Institute for Research and Education, Rio de Janeiro, Brazil

**Keywords:** vaccine impact, narrative review, COVID-19, vaccination campaign, populational studies

## Abstract

**Background:**

The lack of precise definitions and terminological consensus about the impact studies of COVID-19 vaccination leads to confusing statements from the scientific community about what a vaccination impact study is.

**Objective:**

The present work presents a narrative review, describing and discussing COVID-19 vaccination impact studies, mapping their relevant characteristics, such as study design, approaches and outcome variables, while analyzing their similarities, distinctions, and main insights.

**Methods:**

The articles screening, regarding title, abstract, and full-text reading, included papers addressing perspectives about the impact of vaccines on population outcomes. The screening process included articles published before June 10, 2022, based on the initial papers’ relevance to this study’s research topics. The main inclusion criteria were data analyses and study designs based on statistical modelling or comparison of pre- and post-vaccination population.

**Results:**

The review included 18 studies evaluating the vaccine impact in a total of 48 countries, including 32 high-income countries (United States, Israel, and 30 Western European countries) and 16 low- and middle-income countries (Brazil, Colombia, and 14 Eastern European countries). We summarize the main characteristics of the vaccination impact studies analyzed in this narrative review.

**Conclusion:**

Although all studies claim to address the impact of a vaccination program, they differ significantly in their objectives since they adopt different definitions of impact, methodologies, and outcome variables. These and other differences are related to distinct data sources, designs, analysis methods, models, and approaches.

## 1. Introduction

Since 2020, epidemiological studies related to the effects of vaccination against COVID-19 have been gaining prominence in leading international journals, reaching more than 700 studies in the Scopus database in June 2022. These papers apply distinct study designs and address different measures of vaccine performance. Clinical trials first stood out in the search to present the efficacy of the vaccines during their phase-3 periods before licensing for application in the general population.

With the beginning of the vaccination roll-outs worldwide, several researchers were dedicated to evaluating the vaccine’s effects on individuals or populations. Vaccine efficacy is determined by randomized controlled trials, and vaccine effectiveness is estimated from post-introduction observational studies. While effectiveness and efficacy of vaccination measure the direct effect of a vaccine on the vaccinated individuals and aim to describe an individual’s risk reduction after vaccination, studies on vaccine impact address the outcome of a vaccination program in a community. These studies are typically ecological or modeling analyses that compare disease outcomes from pre- and post-vaccine introduction. The reductions in disease outcomes are estimated through the direct effects of vaccination in vaccinated participants and indirect effects due to reduced transmission within a community (1).

Most vaccination efficacy studies assess an individual’s risk reduction after being vaccinated compared with those unvaccinated, thus inevitably addressing vaccine effectiveness (2–4). Vaccination impact studies are typically more feasible since individualized data are not always available in many scenarios. Only aggregated or deidentified data about the vaccination progress is often publicly available to infer how the vaccination roll-out impacts the population. While vaccine effectiveness studies are more consistent in study design and estimates (5), the existing impact studies differ significantly in many perspectives, including different study designs, estimated community outcomes, confounder variables, data sources, methods and models.

Moreover, there are literature works that address the impact of COVID-19 vaccination but should be characterized as vaccine effectiveness studies instead. For example, while the title of the work by Pritchard et al. (6) mentions vaccine impact, it presented the reduction of individual infections in vaccinated people. In the same way, the main results shown in Tande et al. (7) refer to the relative risks between vaccinated and non-vaccinated individuals, and Moghadas et al. (8) presented a theoretical simulation addressing individual outcomes.

The lack of precise definitions and terminological consensus leads to confusing statements from the scientific community about what a vaccination impact study is. In addition, there is a myriad of possible study designs in the literature that address the impact of vaccination programs on distinct populations. Difficulties in comparing study results reduce the understanding of the potential impact of a COVID-19 vaccination program on a specific population.

The present work presents a narrative review describing and discussing COVID-19 vaccine impact studies, mapping their relevant characteristics, such as study design, approach and outcome variables, while analyzing their similarities, distinctions, and main insights. Our search approach not only aims to make explicit the real distinction between vaccination impact studies and vaccination efficacy and effectiveness studies, but also presents a range of possibilities of scope and methods, among other variables. The methodology applied here, followed by other recent publications (9–13), can be used to explain the impact of a vaccination roll-out in a community, guiding and equipping other researchers interested in the subject.

## 2. Materials and methods

We performed the electronic search using the PubMed and Google Scholar databases. The search included literature published before November 30, 2021, using the keywords “covid-19,” “SARS-CoV-2,” “vaccine*” and “impact*.” The articles’ screening included studies addressing the impact of COVID-19 vaccines on population outcomes. The study selection was conducted by: (i) formulating the eligibility criteria; (ii) reading the abstract and selecting for full-text reading; (iii) reading the full-texts and selecting for study inclusion; and (iv) conducting a snowballing process including other studies by forward and backward search (14–17).

We considered the following eligibility criteria for study inclusion: articles covering the vaccine impact research topic, with a design of statistical modeling and/or comparison between pre- and post-vaccination population, and written in English. We excluded articles not covering the topic of COVID-19 vaccination impact, lacking a detailed description, using unclear methods, addressing vaccine effectiveness rather than impact, or related to vaccine acceptance and health impacts. Among those who focused on the vaccine’s effect on preventing COVID-19 cases, most presented the impact from the individual perspective of those vaccinated (effectiveness) and not the effect of the vaccination process on the entire population (vaccine impact). We further conducted a snowballing process from the first set of articles, including the literature before June 10, 2022. The snowballing search is a backward and forward screening, looking at the reference list of the included articles (backward) and the papers citing the included studies (forward) (18). We extracted the following research characteristics to be discussed: author, year, country, period of analysis, data source, design, outcomes and methods/models/approaches applied.

## 3. Results

**Figure 1:**
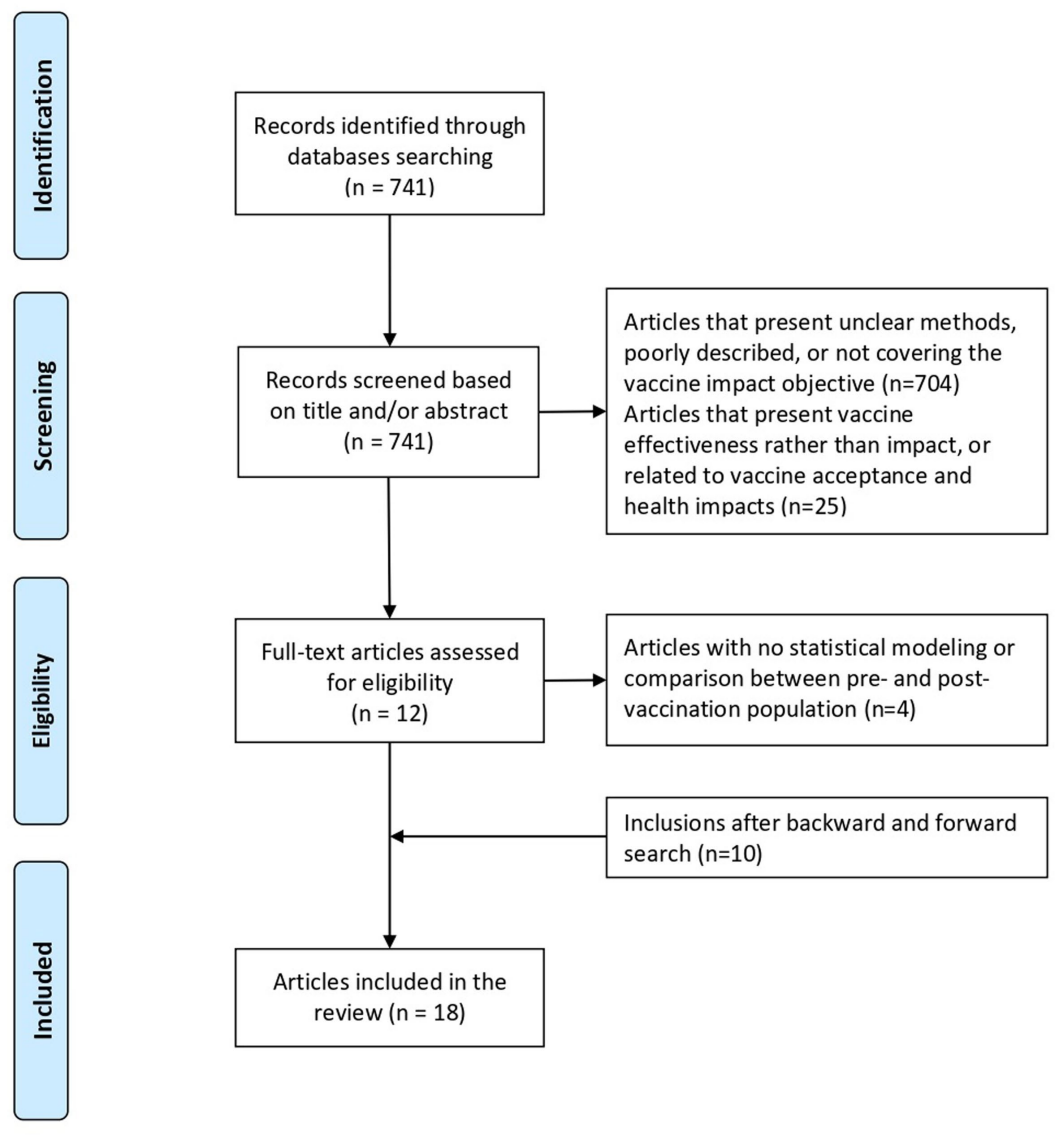
Flowchart of the screening process.

The database search found 741 articles, which were screened following the eligibility criteria. From the title and abstract reading, we excluded 704 papers presenting unclear methods lacking a detailed description or not covering the topic of COVID-19 vaccination impact, and 25 studies analyzing vaccine effectiveness rather than vaccine impact. This first study selection process yielded a set of 12 articles. From the full-text reading, we excluded four papers that did not perform statistical modelling or comparisons between pre- and post-vaccination, leaving a total of eight articles. From the selected papers, we further conducted a snowballing search, finding 269 additional articles citing or being cited by those that composed the initial set. These additional articles were screened with the criteria described before, and 10 new articles were selected, totaling 18 articles. Figure 1 illustrates the flowchart of the whole screening process. As the objective was not to conduct a systematic review, articles with similar approaches covering the same countries whose titles did not cover the vaccine impact theme were not analyzed.

The selected papers presented a diversity of methods, models, and approaches to address the impact of vaccines. Table 1 presents the characteristics of each article in terms of country, period, data source, design, outcomes and methods/models/approaches. The periods of reference for most studies involve 5 months or more. In some cases, the periods of reference for the used data start before the beginning of the vaccination to assess the evolution of cases before and after vaccination. There are studies from Latin America (Brazil and Colombia), North America (United States), Europe (England, Italy, Portugal, and other European countries) and Asia (Israel).

**Table 1:** Main characteristics of included studies.

**Author, year**	**Country**	**Period**	**Data sources**	**Study design**	**Outcomes**	**Method/models/approaches applied**
Cot et al., 2021	US	Dec 2020–Mar 2021	The OpenSky COVID-19 Flight Dataset and Opendatasoft	Epidemiological statistical modeling	COVID-19 case incidence	Epidemic Renormalization Group (eRG)
Mesle et al., 2021	World Health Organization (WHO) European Region	Dec 2020–Nov 2021	The European Surveillance System (TESSy)	Comparison of pre- and post-vaccination population	Averted COVID-19 deaths	Estimation of averted events
McNamara et al., 2022	US	Nov 2020–Apr 2021	Centers for Disease Control and Prevention (CDC)	Comparison of pre- and post-vaccination population	COVID-19 deaths	Difference-in-differences framework
Victora et al., 2021	Brazil	Jan–May2021	Brazilian Ministry of Health System	Comparison of pre- and post-vaccination population	COVD-19 mortality rate	Calculus of COVID-19 age-specific mortality rates
Rossman et al., 2021	Israel	Aug 2020–Feb 2021	Israeli Ministry of Health	Comparison of pre- and post-vaccination population	COVID-19 cases, hospitalizations, and severe hospitalizations	Temporal changes in weekly numbers of several clinical measures
Galvani et al., 2021	US	Oct 2020–Jun 2021	Centers for Disease Control and Prevention (CDC)	Epidemiological statistical modeling	Averted COVID-19 hospitalizations and deaths	Estimation of averted events
Andrews et al., 2021	England	Dec 2020–Mar 2021	Centers for Disease Control and Prevention (CDC)	Epidemiological statistical modeling	Averted COVID-19 hospitalizations and deaths	Estimation of averted events
Machado et al., 2022	Portugal	Dec 2020–Jul 2021	Portugal Health General Office	Epidemiological statistical modeling	Dynamics of confirmed cases and transmissibility index value (Rt)	SEIR model
Haas et al., 2022	Israel	Dec 2020–Apr 2021	National surveillance data from the Israeli Ministry of Health	Comparison of pre- and post-vaccination population	Averted SARS-CoV-2 infections and COVID-19- hospitalizations, severe hospitalizations, and deaths.	Estimation of averted events
Milman et al., 2021	Israel	Dec 2020–Mar 2021	Maccabi Healthcare Services database	Comparison of pre- and post-vaccination population	Relative changes in positive test fraction according to changes in the fraction vaccinated	Correlation analysis
Miłobedzki, 2022	European Union countries	Jan–Jul 2021	Our World in Data	Epidemiological statistical modeling	COVID-19 mortality	Estimation of confirmed new deaths based on infections and vaccinations
Liu et al., 2021	13 middle-income countries (MICs) of Europe.	Mar–Nov 2021	WHO Strategic Advisory Group of Experts on Immunization (SAGE) dataset	Epidemiological statistical modeling	COVID-19 mortality	Transmission Dynamic Model (adapted CovidM)
Caetano et al., 2021	Portugal	Jan–Sep 2021	ACSS/SPMS hospitalization registry	Comparison of pre- and post-vaccination population	COVID-19 averted deaths; Vaccine Effectiveness	SEIR model
Rojas-Botero et al., 2022	Colombia	Mar–Dec 2021	PAIWEB information system of the Ministry of Health and Social Protection	Comparison of pre- and post-vaccination population	COVID-19 averted deaths	Estimation of averted events; Estimation of Vaccine Effectiveness
Sacco et al., 2021	Italy	Jan–Sep 2021	Case- based national COVID-19 integrated surveillance system	Comparison of pre- and post-vaccination population	COVID-19 averted cases, hospitalizations, ICU admissions and deaths; Vaccine Effectiveness	Estimation of averted events; Estimation of Vaccine Effectiveness
Mattiuzzi et al., 2021	Europe (different countries)	Dec 2020–Nov 2021	Data of Meslé et al. (20)	Epidemiological statistical modeling	Association between the percentage of averted deaths of older people and percentage of vaccine uptake in each corresponding European country	Spearman’s correlation and multiple linear regression
Shoukat et al., 2021	US	Dec 2020–Jul 2021	Centers for Disease Control and Prevention (CDC)	Epidemiological statistical modeling	Averted COVID-19 hospitalizations and deaths	Age-stratified agent-based model of COVID-19
Suthar et al., 2021	US	Dec 2020–Dec 2021	Centers for Disease Control and Prevention (CDC)	Epidemiological statistical modeling	COVID-19 case incidence and mortality	Generalized linear mixed models

### 3.1. Summary of studies

Although the selected studies address the impact of a vaccination program, they differ significantly in their objective since they adopt different definitions of impact and methodologies. For instance, some studies have compared COVID-19 outcomes during different periods of the pandemic roll-out (specifically, the pre- and post-vaccination). In contrast, other studies perform a counterfactual analysis to calculate the vaccination program’s impact on a population, estimating what could have been the COVID-19 outcome if either no vaccination program existed, or vaccination uptake had lower levels on the studied population.

Cot et al. (19) built an epidemic Renormalization Group (eRG) framework to reproduce and predict the diffusion of the pandemic in the U.S., taking human mobility across the U.S. and the influence of social distancing into account. Human mobility is monitored using open-source flight data among U.S. states. The eRG framework provides a single first-order differential equation that describes the time-evolution of the cumulative number of infected cases in an isolated region. Meslé et al. (20) estimated the number of deaths directly averted in the population of older adults (60 years and older) due to COVID-19 vaccination in the WHO European Region from December 2020 to November 2021. The authors simulated COVID-19 outcomes in a scenario without vaccination. The simulation parameters were based on information from previous studies of COVID-19 vaccine effectiveness in preventing deaths, thus calculating the number of directly averted deaths for each country. The analysis also applied an adapted formula used by Machado et al. (21) to measure the influenza vaccine program impact, which calculates the number of deaths averted with one dose and with full vaccination through two different equations. The equations associate death numbers with vaccine effectiveness and vaccination uptake.

McNamara et al. (22) estimated the national-level impact of the initial phases of the COVID-19 vaccination program in the US. The authors compared relative changes in four different outcomes considering pre- and post-vaccination periods for the whole population and age groups. The authors applied a difference-in-differences framework to evaluate whether outcomes declined rapidly after vaccination roll-out in age groups with earlier vaccine eligibility. McNamara et al. (22) is mentioned by Ortiz and Neuzil (1) as an example of a COVID-19 vaccination program impact study. Victora et al. (23) investigated whether vaccination impacts the mortality of older individuals in a context of SARS-CoV-2 gamma variant (P.1 lineage) dominance in Brazil. The study analyzed the changes in COVID-19 proportionate mortality and mortality rate ratio in different age groups during the increase of vaccination coverage. First, they obtained proportionate mortality for older individuals (i.e., the ratio between the number of COVID-19 deaths at ages 70–79 and 80+ years and total number of COVID-19 deaths). Second, they calculated COVID-19 age-specific mortality rates by dividing the numbers of weekly deaths by the estimated population by age group. Mortality rates at ages 70–79 and 80+ years were then divided by rates for the age range 0–9 years in the same week, resulting in mortality rate ratios.

Rossman et al. (24) analyzed the temporal dynamics of new COVID-19 cases and hospitalizations after the vaccination campaign to distinguish the possible impact of vaccination from other factors, including a third lockdown implemented in Israel in January 2021. The authors performed several comparisons: individuals aged 60 years and older were prioritized to receive the vaccine first versus younger age groups; the January 2021 lockdown versus the September 2020 lockdown; and early vaccinated versus late-vaccinated cities. Galvani et al. (25) estimated the impact of the US COVID-19 vaccination campaign in controlling the virus’s transmission and deaths. The authors compared COVID-19 outcomes on the current scenario with two counterfactuals: 50% of vaccination coverage and without a vaccination campaign. They estimated the averted number of COVID-19 deaths and hospitalizations, and calculated the adjusted odds ratios for vaccination impact, stratified by vaccine platform and previous SARS-COV-2 infection. To evaluate the vaccination program impact in the US, the researchers expanded their COVID-19 age-stratified agent-based model to include transmission dynamics of the different variants. They also used the population demographics, the contact network accounting for pandemic mobility patterns, and age-specific risks of severe health outcomes due to COVID-19 as model parameters.

Andrews et al. (26) estimated the number of deaths prevented by vaccination in England between the start of the vaccination program and the end of March 2021. Assessments are made to compare the COVID-19 mortality in the current scenario with an estimated counterfactual scenario without a vaccination program. Machado et al. (27) analyzed the impact of vaccination on the control of the pandemic. They investigated the relationship between vaccine coverage and non-pharmacological interventions (NPIs), developing different scenarios for the fade-out of NPIs as vaccine coverage increases in the population. The analysis is based on developing a standard mathematical model for assessing the population-level impact of a COVID-19 vaccine in a community. A SEIR model is created by splitting the total human population into mutually exclusive compartments: unvaccinated susceptible vaccinated, susceptible, early exposed, pre-symptomatic infected, symptomatically infected, asymptomatically-infected, hospitalized and recovered.

Haas et al. (28) analyzed the number of averted COVID-19 infections, hospitalizations, and deaths in Israel due to the nationwide vaccination campaign using the Pfizer-BioNTech BNT162b2 mRNA COVID-19 vaccine. The authors estimated the direct effects of the immunization program for all susceptible individuals who were at least with one dose of COVID-19 vaccine compared to unvaccinated individuals. Moreover, Milman et al. (29) analyzed the community-level evidence for SARS-CoV-2 vaccine protection of unvaccinated individuals using a correlation analysis to test results collected during the rapid vaccine rollout in a large population from 177 Israeli communities. To control for the spatiotemporally dynamic nature of the epidemic, they focused on relative changes in the proportion of positive tests within each community between fixed time intervals.

Miłobedzki et al. (30) estimated the number of confirmed new deaths based on infections and vaccinations for the European Union countries. They computed the long-run marginal death effect concerning confirmed infections and compared it with respect to confirmed vaccinations. The authors also calculated the minimal weekly number of new vaccinations per million population in a European country to keep the number of new deaths per million population at a certain level. Liu et al. (31) applied a dynamic transmission model to analyze possible dosing interval strategies for two-dose COVID-19 vaccination in thirteen European middle-income countries and compared their impacts in terms of mortality. A vaccine with similar characteristics to AstraZeneca (AZD1222) was used in the base scenario. The authors also included sensitivity analyses considering different values for vaccine efficacy.

Caetano et al. (32) estimated the COVID-19 averted deaths in Portugal using a SEIR model to measure the impact of vaccination strategy. The authors adapted an age-structured SEIR deterministic model and used hospitalization data for the model calibration to measure the impact of the COVID-19 Portuguese vaccination strategy on the effective reproduction number. They also explored three scenarios for vaccine effectiveness waning: the no-immunity-loss, 1-year and 3-year immunity duration scenarios. Rojas-Botero et al. (33) estimated the number of directly averted deaths due to COVID-19 vaccination among older adults in Colombia. The authors calculated the full vaccination coverage of older adults, for each epidemiological week and age group, from March to December 2021. A sensitivity analysis considered variations in vaccine effectiveness by age group. Sacco et al. (34) estimated the number of averted COVID-19 cases, hospitalizations, intensive care unit admissions, and deaths by COVID-19 vaccination in Italy. The authors applied a method widely used in the study of vaccination impact during the influenza season (21, 35).

Mattiuzzi et al. (36) measure the association between the percentage of averted deaths of older people and the percentage of vaccine uptake in each corresponding European country. The authors used data on vaccine uptake and efficacy to perform univariate (Spearman’s correlation) and multivariate (multiple linear regression analysis) correlations to determine the association of the percentage of averted deaths with vaccine uptake and the type of vaccine administered. Shoukat et al. (37) applied an age-stratified agent-based model of COVID-19 in US data to estimate the averted COVID-19 hospitalizations and deaths due to the vaccination roll-out. The model was calibrated using reported incidence in New York City (NYC), considering the relative transmissibility of each variant and vaccination coverage. The authors simulated the COVID-19 outbreak in NYC under the counterfactual scenario of no-vaccination and compared the resulting disease burden using the number of cases, hospitalizations, and deaths reported under the actual vaccination status. Also in US, Suthar et al. (38) used generalized linear mixed models assuming a negative binomial outcome distribution to analyze the impact of vaccines in reducing COVID-19 incidence and mortality. The authors also included a first-order autoregressive correlation structure to account for multiple observations per municipality and to identify potential autocorrelation.

The set of studies herein described sought to establish causal relationships between the vaccination process and different outcomes related to COVID-19. However, in Cot et al. (19) and Rossman et al. (24), there is the intermediation of confounders variables such as mobility and non-pharmacological interventions (NPI). Studies based exclusively on simulations, such as the one from Iboi et al. (39), were not included. Although all studies aimed to estimate the impact of the vaccination roll-out in a population-level, they used different analysis methods, which implies diverse models and tools, to achieve their established objectives. For instance, while Meslé et al. (20) estimated vaccination campaigns’ impact by calculating the number of averted deaths, the study by McNamara et al. (22) estimated by comparing pre-vaccination COVID-19 outcomes with post-vaccination outcomes. In this sense, the study by Fang et al. (40) used the association between the vaccination coverage and the incidences and deaths caused by COVID-19 to calculate the impact of each percentage increase in population vaccination rates in the reduction of county-wide COVID-19 incidence and mortality. Often, the analysis method explained how the explanatory and outcome variables were associated. The differences among the analysed studies regarding their objectives lead to significant contrasts in the analysis methods, tools, and variables considered.

### 3.2. Data sources

The most important data for the studies are those related to the COVID-19 vaccination campaign, the confirmed cases and their outcomes. Usually, the National Ministry of Health and the Centers for Diseases Control are the main sources of these data. Nonetheless, depending on the approaches applied, other sources (secondary data) are also considered, as in Mattiuzzi et al. (36), which used the data produced by Meslé et al. (20).

### 3.3. Study design

By analyzing the populational level of the data used in the studies and their observational nature, we can say that all the studies follow an ecological study design, according to Levin et al. (41). More specifically, and according to Hanquet et al. (42), the impact of a vaccination program is estimated by comparing the population with access to a vaccination program with a reference population without the program, and vaccination program impact studies may follow mainly three different designs, which are specific subtypes of an ecological study:

- *Comparison of pre- and post-vaccination population.* According to this design, the two populations being assessed are separated by time, and the study outcome is compared between the pre- and post-vaccination periods. In this design, it is important to consider the different control measures (or non-pharmaceutical interventions) imposed by governments to the population being analysed in these two periods. Some initiatives such as the Oxford Covid-19 Government Response Tracker – OxCGRT (43) systematically collects daily data on policy measures enforced by governments (e.g., school closures, travel restrictions, vaccination policy, lockdowns) to tackle COVID-19 since the beginning of the pandemic and across more than 180 countries, and define indicators which may help leverage the impact of a vaccination program taking into account the different stringency levels applied to the pre- and post-vaccination populations.
- *Cluster randomized vaccination trials.* This design is based on generating comparable social units called clusters by randomization. The outcome is compared between placebo and vaccine clusters. Cluster-randomized trials are usually conducted to quantify a treatment or intervention effect. In cluster-randomized trials, individuals are grouped based on specific characteristics (e.g., neighbourhood of residence), and the entire cluster is randomized to treatment or control. The process of randomization ensures that the treatment and control groups are exchangeable. This approach is useful when it is impractical or infeasible to randomize at the individual level. The randomized clusters can be compared to assess the overall impact of an intervention, which is particularly important in settings where intervention may have indirect effects (44).
- *Statistical modelling.* This design is normally associated with an outcome prediction (e.g., disease occurrence) without vaccination. It compares it to the population’s occurrence with vaccination programs, henceforward named “epidemiological statistical modelling.” This design can adjust for differences between populations, such as annual variations and secular disease trends or changes in health care use.

As shown in Table 1, none of the analysed articles followed the cluster-randomized vaccination trial study design. This is possibly due to the urge brought by the pandemic to vaccinate the worldwide population with vaccines which effectiveness has already been attested (45).

Nine out of the eighteen studies followed the epidemiological, statistical modelling study design, aiming to predict the impact on a community outcome by simulating scenarios with and without a vaccination roll-out. Meslé et al. (20), Galvani et al. (25), and Andrews et al. (26) estimated the number of either averted deaths or averted hospitalisations or both. To make these estimations possible, vaccine efficacy and effectiveness against deaths and hospitalisations studies were considered input variables of the impact study. In particular, Meslé et al. (20) proposed a standard approach to compare the estimated direct impact of the differential roll-out of COVID-19 vaccination programs across 33 countries in the WHO European Region, from December 2020 to November 2021. They calculated the weekly number of deaths averted per country taking the number of confirmed cases, vaccine coverage, and vaccine effectiveness in the given locality and time range into account, following Machado et al. (21). They also differed the vaccine coverage and effectiveness with at least one dose (which they called VU1 and VE1) from the vaccine coverage and effectiveness for those with complete vaccination schemas (VU2 and VE2), understanding that the number of vaccine doses influences the development of a full immune response individually, and consequently the protection from severe infection and death. Lower and upper bounds used for VE1 and VE2 were chosen based on observational studies for the vaccines most frequently used in the countries of that study. In their study, Meslé et al. (20) confirmed that both speed and extent of the vaccination in some eligible groups were determinants of vaccination impact with regard to averted deaths. Galvani et al. (25) also acknowledged the effectiveness of the different COVID-19 vaccine types administered in the US from October 2020 to June 2021 in preventing severe diseases, hospitalizations, and deaths due to COVID-19, which in turn contributed to increasing the impact of the vaccination program, potentially because of the vaccine’s ability to reduce transmission of the virus.

The remaining studies followed the pre- and post-vaccination population comparison design. In McNamara et al. (22) and Rossman et al. (24), there were clear rules to define when a specific age group goes from pre-vaccination status to post-vaccination status. However, there is no such specification in Victora et al. (23), which analyses COVID-19 community outcomes over time, while vaccination coverage rises for the age groups studied.

Meslé et al. (20) applied the same formula to measure the averted deaths due to vaccination for all the populations from the 33 countries covered by their study. Even though all analyzed countries are from the WHO European region, they differ in many aspects, including geographical, sociodemographic, and vaccination programs, since each country applied vaccines from different manufacturers, which is even pointed out in the study. Moreover, the analysis described by Meslé et al. (20) also assumes that populations with and without vaccination programs (a.k.a. pre- and post-vaccination populations) have similar baseline transmission (hence the clustered populations are similar), which does not hold. Milman et al. (29) presented the relative change in the positive test fraction according to the change in the proportion of vaccinated individuals. Finally, data completeness is also essential for ecological studies. Complete and accurate data is fostered in the different health systems, but huge variation in quality and validity remains across organizations (46).

### 3.4. COVID-19 community outcomes analyzed

Although all articles address the impact of vaccination programs, the outcomes differ significantly. The results from the studies compare the dynamics of the pandemic based on different outcomes, with or without an ongoing vaccination program, and even simulating different vaccination scenarios. They calculate the variations in the disease outcome, which may refer to the reduction in cases, hospitalizations, deaths, or the number of deaths averted. It is important to note that a comparative analysis between studies is hampered by the different ways the impacts of vaccination processes are presented. Most studies estimated the impact in terms of averted COVID-19 deaths (20, 25, 26, 28, 32–34, 36, 37), and some of them also analyzed the averted hospitalizations (and severe hospitalizations) (24–26, 28, 34, 37). Other studies investigated the COVID-19 incidence seeking to estimate the reduction in the number of cases (19, 21, 28, 29, 34, 38).

There could be a more specific interest in low- and middle-income countries (LMICs) in view of the considerable obstacles in both receiving and distributing doses, especially at the beginning of the vaccination roll-out when vaccines were scarcer. In the three studies related to countries with disparities in access to healthcare and potential discrimination in vaccine distribution, the results are in line with those of developed countries in terms of impact. In both South American countries (Brazil and Colombia) and thirteen European countries (Albania, Armenia, Azerbaijan, Belarus, Bosnia and Herzegovina, Bulgaria, Georgia, Republic of Moldova, Russian Federation, Serbia, North Macedonia, Turkey, and Ukraine), they successfully adopted strategies based on staggering vaccination in age groups, prioritizing older adults. All studies point to significant and relevant impacts of vaccine campaigns on the analyzed populations, whether due to the variation in the proportion of deaths in different age groups, the declines observed for the prioritized groups in the curves of cases and deaths, or the number of deaths avoided. The findings of each study are presented in Supplementary Table S1.

## 4. Discussion

The impact studies included in the present narrative review show significant differences in how they are developed and the main achieved outcomes. The analysis methods and tools are also quite different. We only selected articles based on actual vaccination data (even if combined with hypothetical vaccination scenarios) and those presented due to vaccine impact on the entire population. The selected studies covered European, Latin American, North American and Asian countries. The reviewed studies used data collected between December 2020 and June 2022.

Most COVID-19 vaccination campaigns worldwide have multiple vaccine platforms available to immunize a population. Therefore, the vaccination impact is not often associated with a vaccine from a single manufacturer. However, Israel exclusively used the Pfizer-BioNTech BNT162b2 mRNA COVID-19 vaccine. Thus, works from Rossman et al. (24), Milman et al. (29) and Haas et al. (28) could address the impact of a single platform vaccination campaign.

Cot et al. (19) established the relationship between the weekly percentage of the vaccinated population and the number of infections. The number of deaths averted by the vaccine is the main result of Meslé et al. (20), covering 33 European countries. Works from Rossman et al. (24), Andrews et al. (26), Galvani et al. (25), and Victora et al. (23) make use of temporal differences in the vaccination rate of different age groups to show a reduction in deaths, contamination, and/or hospitalizations for distinct age groups.

Regarding confounders, Rossman et al. (24), Andrews et al. (26), Galvani et al. (25), and Victora et al. (23) adjusted their results by age group. In Galvani et al. (25), the mobility rate was considered in the model. Notably, Victora et al. (23) and Galvani et al. (25) mentioned different variants of concern (VoCs) of the SARS-Cov virus; however, these VoCs should not be characterized as confounders of these studies since they were not explicitly taken into account in the models. Thus, pre- and post-vaccination populations were assumed to have similar baseline transmission. These studies only mentioned the VoCs that were dominant in the studied populations: Victora et al. (23) study was conducted when gamma was the dominant VoC, while Galvani et al. (25) was conducted during the dominance of the Alpha, Gamma, Delta, and the original Wuhan-1 variants. Likewise, vaccine manufacturers were not explicitly addressed in the models to calculate the impact and should not be considered confounder variables.

The studies also differ in outcomes, involving deaths, hospital admissions, incidences, non-ICU hospitalizations, ICU hospitalizations, and symptomatic cases. Machado et al. (27), Cot et al. (19), and Rossman et al. (24) addressed the impact of other interventions or occurrences used as parameters; the following interventions or occurrences were mentioned: pre-existing immunity, self-isolation of infected individuals, state stay-home order, state facemask police or proportions of members of public who wear masks in public and, finally, lockdowns. Rossman et al. (24) and Machado et al. (27) used the impact of lockdown as a model parameter.

The way of presenting the results is also quite different. Most articles present the number of cases, hospitalizations or deaths averted. In some studies, the asymmetry in the temporality of the vaccination process between different age groups is used to point out how it affects the relative participation of these groups in the total number of cases, deaths or hospitalizations. There are approaches that establish comparisons between countries and territories. In these cases, the different vaccination rates observed are related to different declines in the numbers of cases, hospitalizations or deaths. There is a specific article that studies the differentiated impact determined by the different intervals between doses. Finally, there is a study in which the authors identify the minimum weekly vaccination rate to guarantee a specific value for the number of deaths.

Several of the reviewed articles made use of epidemiological models such as SEIR, which can be seen as simplistic, that is, using a few compartments instead of thousands of compartments to represent the real complexity of the system. In an epidemic, several phenomena are difficult to understand, caused by the interaction of a huge number of agents which, even when acting locally, are capable of influencing results elsewhere (47). However, SEIR models proved robust enough to be applied in different geographic locations and in populations of different ethnic origins, enabling and recommending their use in territories with a lack of viral testing. Barbosa et al. (48) applied the SEIR model using epidemiological data from Marabá, a poor municipality in the state of Pará in Brazil, with estimated values of latency time and infectious time obtained in Chinese populations and these proved to be useful for predicting the evolution of COVID-19 cases, a more complex process than the estimation of the vaccine impact. One last important consideration concerning the presented models is that they do not recognize the prolonged duration of the pandemic and the representative rate of deaths during a given period, since they do not use the number of deaths and births in the most active period of the pandemic as parameters, thus, disregarding its vital dynamics, foreseen in the complex systems applied to epidemiological models presented by Lima (47).

**Figure 2:**
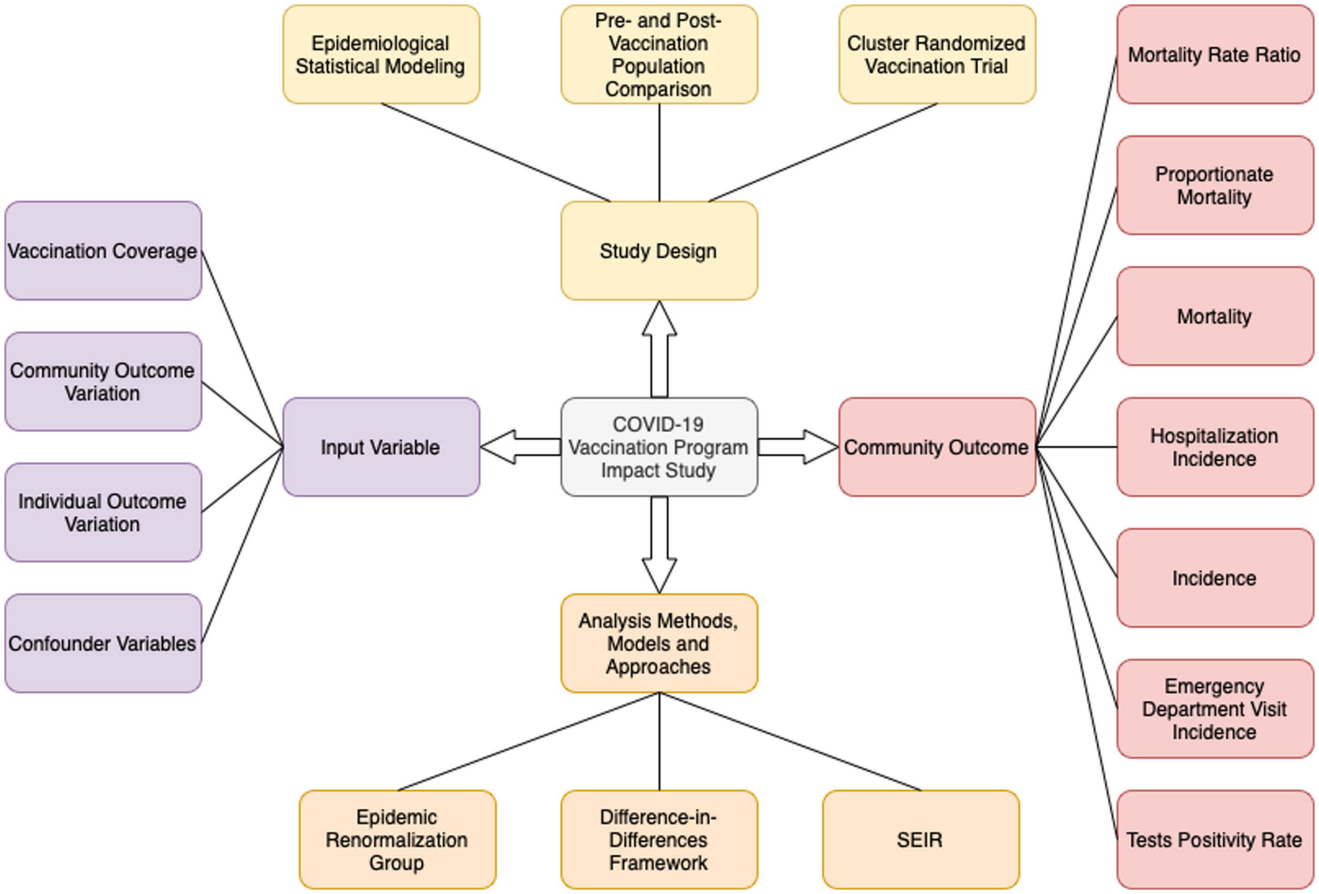
The main characteristics of the vaccination impact studies analysed in this review. As visually represented, a vaccination impact study is distinguished by four perspectives: Input variables, Community outcome, Study design, and Analysis methods, models, and approaches.

Figure 2 synthesizes the main characteristics of the vaccination impact studies analyzed in this narrative review. Although the lack of systematicity in the review process does not allow the complete range of designs, methods, variables, and results of vaccine impact studies to be presented, it is understood that the outline of its characteristics is broad enough to give those interested in the subject the breadth of possibilities available.

## 5. Conclusion

The articles analyzed in this Narrative Review, regardless of the methods applied and country(ies) covered, share in their results the significant population impact brought by the vaccination process. Although the pandemic is cooling down at the moment, its permanence has required new booster doses to be administered. In addition, the possibilities for the emergence of new variants of concern can alter vaccine efficacy, establishing new levels of vaccine impact. Studying the impact of COVID-19 vaccines will remain the slogan of the day for some time. The present work contributed to the research on this theme, offering a broad and structured view of the methodological possibilities, models, approaches and designs. Furthermore, it aims to contribute to a broader view of the possible studies, as it also brings together the different possibilities of input variables adopted and different outcome variables that may represent the vaccination impact.

There is, however, an approach to vaccine impact that remains underexplored. In addition to disparities in the application of COVID-19 tests and in the supply of protective equipment, LMICs suffered from problems related to the availability of vaccines (49). In none of the analyzed studies, the discussion on the superiority of the strategy, adopted by the richest countries, in terms of vaccine impact, of protecting them-selves other than globally controlling the COVID-19 pandemic, was privileged. In one of the studies, Louden (50) says that careful consideration of vaccine production, pricing, allocation, and distribution must be taken into account to ensure equitable access to COVID-19 vaccines scaling up the global COVID-19 vaccination program but in this study vaccine impact was not the approach. Ali et al. (51) discussed the problem of vaccine equity in LMICs. They found that inequalities in wealth, education, and geographic access can affect vaccine impact and vaccination dropout which demands more attention in countries where the level of inequality is considerably higher. The analysis of global vaccination rollouts comparing LMIC to rich countries should include each country demography (and the age groups approved to be vaccinated). LMICs with low proportion of population older than 60 years cannot be direct compared to some European countries with high proportion of population older than 60 years. Furthermore, the propensity of young adults to get vaccinated in a country with a young population is different than in one with an old population. The assessment of the benefits of potentially protecting older adults to the risks of the vaccine is dependent on demography.

Another study alternative, which was not observed, would be to evaluate the vaccine impact in terms of the relative dynamics of cases and deaths after the consolidation of the vaccination process, comparing the results between countries before and after vaccination, as a function of the percentage of vaccinated and the number of doses administered. Brazil, for example, a LMIC, due to the greater adherence of its population to vaccination, after being the eighth country in deaths by COVID-19 in the world in the period of 2020–2021, already occupies the 17th position in October of 2022, considering the entire pandemic period, registering fewer deaths *per capita*, bearing in mind only the year 2022, than the United States and many European developed countries. Finally, the impact of vaccination campaigns could be analyzed in terms of compliance with COVID-19 regulations, mobility and contact behavior in communities, the likelihood of transmission at a potentially infectious contact, human behavior (and mental health), and health care costs.

## Author contributions

HF, LM, FBa, and IP: conceptualization, formal analysis, investigation, and visualization. HF, LM, FBa, and IP: methodology and writing—original draft preparation. FBa, IP, OR, SH, PM, and FBo: validation. HF, LM, and IP: data curation. IP, OR, SH, PM, and FBa: writing—review and editing. OR, SH, and FBo: supervision. All authors contributed to the article and approved the submitted version.

## Funding

This work was performed as part of the Grand Challenges ICODA pilot initiative (INV-017293), funded by the Minderoo Foundation and the Bill & Melinda Gates Foundation. The research was also supported by the National Council for Scientific and Technological Development (CNPq) [grant numbers 310940/2019-2 and 403863/2016-3 to SH; 422810/2021-5 and 312059/2022-1 to FBa; and 422470/2021-0 and 311519/2022-9 to PM], Carlos Chagas Filho Foundation for Research Support of the State of Rio de Janeiro (FAPERJ) [grant numbers 211.308/2019 to FBa; and 211.086/2019, 211.645/2021, and 201.348/2022 to PM], and the Pontifical Catholic University of Rio de Janeiro (PUC-Rio). OTR is funded by Sara Borrell from Instituto de Salud Carlos III (CD19/00110).

## Conflict of interest

The authors declare that the research was conducted in the absence of any commercial or financial relationships that could be construed as a potential conflict of interest.

## Publisher’s note

All claims expressed in this article are solely those of the authors and do not necessarily represent those of their affiliated organizations, or those of the publisher, the editors and the reviewers. Any product that may be evaluated in this article, or claim that may be made by its manufacturer, is not guaranteed or endorsed by the publisher.
