# Supplementary material for: What we talk about when we talk about COVID-19 vaccination campaign impact: a narrative review
Source: Front Public Health. 2023 May 11;11:1126461. doi: 10.3389/fpubh.2023.1126461 (PMC10211334; doi:10.3389/fpubh.2023.1126461)
Supplement: Supplementary file 1 [file Table_1.docx]

**Supplemmentary Material 1**

**Table S1. Main findings of included studies**

| **Author, Year** | **Averted deaths** | **Averted**  **hospitalisations** | **Averted**  **infections** | **Other findings** |
| --- | --- | --- | --- | --- |
| [Cot et al., 2021] | - | - | - | The results show that vaccinations alone are not enough and strict social distancing measures are required until suficient immunity is achieved. |
| [Mesle et al., 2021] | 469,186 (129,851 - 733,744) | - | - | - |
| [McNamara et al., 2022] | - | - | - | Deaths declined by 41% (14-69%) among adults aged 65–74 years and by 30% (47-66%) among those aged ≥75 years compared with adults aged 50 to 64 years |
| [Victora et al., 2021] | - | - | - | The proportion of all COVID-19 deaths at ages 80+ years was over 25% in weeks 1-6 and declined rapidly to 12.4% in week 19, whereas proportionate COVID-19 mortality for individuals aged 70-79 years started to decline by week 15. Mortality rates were over 13 times higher in the 80+ years age group than that of 0-69 years old up to week 6 and declined to 5.0 times in week 19. |
| [Rossman et al., 2021] | - | - | - | The reduction in cases and hospitalisations was larger and earlier in individuals 60 years and older than in younger individuals. The decrease in clinical measures was according to the prioritization schedule. A decrease of 45% versus 28% in the percentage of positive tests and 68% versus 22% in hospitalizations was observed in individuals 60 years and older compared to individuals aged 20–39 years. |
| [Galvani et al., 2021] | 279,000 | 1.25 million | - | If the U.S. had achieved only half the actual pace of vaccination, there would be 121,000 averted deaths and 450,000 averted hospitalisations. |
| [Andrews et al., 2021] | 10,400 (aged 60 years or older). | - | - | - |
| [Machado et al., 2022] | - | - | - | The results presented the dynamics of confirmed cases and transmissibility index value (Rt). The results are associated with three different scenarios based on other criteria for implementing non-pharmacological interventions. |
| [Haas et al, 2022] | 5,532 (3,085 - 7,982) | 24,597 (18,942 - 30,252) | 158,665 (144,640 - 172,690) | Averted severe hospitalisations: 17,432 (12,770 - 22,094) |
| [Milman et al, 2021] | - | - | - | On average, for each 20% of individuals who are vaccinated in a given population, the positive test fraction for the unvaccinated population decreased approximately twofold. The results provide observational evidence that vaccination not only protects individuals who have been vaccinated but also provides cross-protection to unvaccinated individuals in the community. |
| [Miłobedzki, 2022] | - | - | - | The long-run marginal death effect with respect to confirmed infections (0.0371) is in absolute terms about 71.62 times greater than that with respect to confirmed vaccinations (−0.000518). |
| [Liu et al., 2021] | - | - | - | In all countries modelled, optimal strategies are those that prioritise the first doses among older adults (60+ years) or adults (20+ years), which lead to dosing intervals longer than six months. In comparison, a four-week fixed dosing interval may incur 10.1% [range: 4.3% - 19.0%; n = 13 (countries)] more deaths. |
| [Caetano et al., 2021] | - | - | - | A significant vaccination coverage of those above five years old, a vaccine effectiveness against disease of at least 80% and softer non-pharmaceutical interventions (NPIs), such as mask usage and social distancing, would be necessary to control disease spread in the worst scenario considered. |
| [Rojas-Botero et al., 2022] | 22,000 (19,597 - 36507) (aged 60 years or older) | - | - | - |
| [Sacco et al., 2021] | 22,067 (13,571 - 48,026) | 79,152 (53,209 - 148,756) | 445,193 (331,059 - 616,054) | Averted severe hospitalisations: 9,839 (6,434 - 16,276) |
| [Mattiuzzi et al, 2021] | - | - | - | A significant linear association was found between the percentage of averted deaths of older people and percentage of vaccine uptake in each corresponding European country (Spearman’s correlation: r=0.872; p < 0.001). In multiple linear regression analysis, the percentage of deaths averted by COVID-19 vaccination remained independently associated with vaccine uptake (p<0.001), but not with the type of vaccine administered (p=0.264). |
| [Shoukat et al., 2021] | 8,508 (7,374 - 9,543) | 48,076 (42,264 - 53,301) | 290,467 (232,551 - 342,664) | - |
| [Suthar et al., 2021] | - | - | - | Every 10% improvement in vaccination coverage was associated with an 8% reduction in mortality rates and with a 7% reduction in case incidence. |
